# First-year university students' self-regulated learning during the COVID-19 pandemic: a qualitative longitudinal study

**DOI:** 10.1007/s11858-022-01444-5

**Published:** 2022-10-24

**Authors:** Michael Liebendörfer, Leander Kempen, Stanislaw Schukajlow

**Affiliations:** 1 University of Paderborn, Paderborn, Germany; 2 TU Dortmund University, Dortmund, Germany; 3 University of Münster, Münster, Germany

## Abstract

When the COVID-19 pandemic began, many universities switched to fully online teaching. This unexpected switching to online teaching was challenging for both teachers and students, and restrictions that were put in place because of pandemic made this challenge even greater. However, new ways of teaching might also open new opportunities for students’ learning. The research question driving our study was as follows: how do students regulate their learning and specifically their choice of resources and peer learning in university mathematics classes that are fully taught online as offered during the COVID-19 pandemic? We report on a longitudinal, qualitative study in which students recorded a brief audio diary twice a week over one whole semester (14 weeks). We focused on three students who completed 70 interviews in total and finished the semester with varying degrees of success. The results show how the students structured their studying (e.g., the roles that deadlines or synchronous teaching events played). They illustrate the strengths and limitations of digital materials provided by the lecturer and the use of complementary media. Further, the pandemic uncovered the double-edged role of simple, often anonymous exchanges (e.g., via Discord servers), with few binding forces for either side, and the significance of stable learning partnerships for students’ success. Our research highlights aspects that should be focal points when comparing traditional instruction and online instruction during the pandemic from a self-regulatory perspective. Practical implications refer to how these aspects can be combined sensibly in fully online courses, but also in blended learning contexts.

## Studying mathematics during the pandemic

The COVID-19 pandemic has strengthened online teaching considerably. The first lockdown in spring 2020 in Germany, as in many places in the world, led to fully online teaching, and students could hardly ever meet in person even for study groups. Teachers had to plan their courses while guessing how students would respond. Thus, in many places, what occurred was what Engelbrecht and Harding had warned about several years earlier: “When starting out on online education, in the absence of the knowledge of what will work and what not and with no real online pedagogy available, many teachers will try to merely convert their traditional courses to the Internet “ (Engelbrecht & Harding, 2005, p. 254).

The aim of this study is to illustrate students’ self-regulated learning when converting traditional courses to the internet. We conducted a longitudinal qualitative study that would be able to provide in-depth insights into the learning processes of three students during their first semester. We analyzed data that were coded from brief, qualitative reports that the students provided twice a week. The results illustrate a well-known theory in the new setting. The longitudinal qualitative view helps in identifying when and why students might regulate their behavior differently in online teaching. This study in particular helps teachers to support students’ choice of resources or effective peer learning.

## Literature review

### Self-regulated learning

Self-regulated learning has been receiving more and more attention in mathematics education since the 1980s. It ties in with constructivist views of learning by assigning learners control and agency over their own learning process (Corte et al., 2000). Students are assumed to be able to control their actions and thoughts at will, and this is crucial for their learning.

This study is based on Boekaert's (2011) dual processing theory, in which it is claimed that students choose their strategy depending on their appraisal of a task and their subsequent goal orientation. Two types of strategies are distinguished. Learning strategies (or problem solving strategies) focus on mastery of the content. These strategies refer to students’ attempts to learn, including peer learning and the choice of resources. In contrast, another type of strategies involves coping strategies that focus on students’ well-being. They are related to students’ high effort but also include copying homework or avoiding tasks.

Goals are important for self-regulated learning of mathematics in general (Goldin et al., 2011; Schoenfeld, 2015) and university mathematics in particular (Schoenfeld et al., 2016). To classify students’ goals, we cluster them according to Pekrun’s (2006) control-value theory (see Göller & Rück, in press, for a similar approach). Pekrun (2006) differentiated between three kinds of goals, as follows. Learning goals are related to gaining knowledge, performance goals are related to demonstrating performance to oneself or others, and well-being goals are related to avoiding threats to the student’s self. Whereas learning goals mostly relate to learning and problem solving strategies and well-being goals mostly relate to coping strategies, performance goals may relate to both types of strategies.

### Self-regulated learning in university mathematics

In mathematics, the secondary-tertiary transition has long been known to be challenging (Gueudet & Thomas, 2020). Attending a university requires students to engage in significantly different learning behaviors than they were used to in high school (Gueudet & Thomas, 2020). Thus, self-regulation is very challenging for first-semester mathematics students, as they feel they have little control over their learning (Göller & Rück, in press).

In Germany, it is common for a university setting to include lectures, tutorials, and compulsory weekly homework. Students often get stuck when trying to solve problems and lack alternative actions for regulating their behavior (Liebendörfer & Hochmuth, 2015). In this contribution, we focus on two strategies that students use often (Göller, 2021) and that may be specifically affected by the pandemic. The first strategy involves the choice of resources. The most important resources are lecture notes, which usually delimit the learning material (Gueudet & Pepin, 2018). Even though more and more digital resources are available, many students prefer classical resources (e.g., textbooks; Rønning, 2014). The second strategy is peer learning. Most students cannot complete all tasks themselves (Rach & Heinze, 2013) and thus form study groups to help them meet the universities’ expectations (Gueudet & Pepin, 2018).

When choosing their strategies, performance goals dominate students’ learning goals. Göller (2021) showed, for example, that students tend to focus on the homework assignments and invest much less time in reviewing lecture content independently of assignments. Because many students feel a great deal of pressure (Liebendörfer & Hochmuth, 2015) and frustration (Liebendörfer & Hochmuth, 2017), protecting their well-being also plays an important role in students’ self-regulation (Göller & Rück, in press).

### Studying mathematics using digital media

Digital media have been used in mathematics education for decades, leading to both opportunities and difficulties (Engelbrecht & Harding, 2005). We focus on the role of digital technology in students’ use of strategies. Previous work has shown that the perspective of self-regulated learning is highly relevant for online learning of mathematics (Adam et al., 2017). Yet, there has been limited research on how strategies such as using resources and peer learning, which are quite broadly defined in psychology, play out in online learning of mathematics.

The flexibility of many offerings in terms of time and place, and the fact that many media can be viewed repeatedly, call for new strategies (Trenholm et al., 2012). Trenholm and Peschke (2020) pointed out that in online education, students need to plan and regulate the amount of new knowledge they consume and the speed with which they consume it, unlike in traditional teaching, where it is the teacher’s duty to regulate these aspects. Students may further need to regulate their effort more actively, for example, by focusing their attention on the content and not on distractions, which are much more prevalent in online teaching (Boz & Adnan, 2017; Trenholm et al., 2012). Accordingly, high-achieving students differ from low-achieving students in their more frequent use of effort regulation (Kim et al., 2015).

With digital resources, the first question is one of choice. When seeking help to complete tasks, students may focus on keywords without paying much attention to the mathematical content (Aguilar & Puga, 2020). However, the more resources are available, the more specific the selection must be (Kempen & Liebendörfer, 2021; Rønning, 2014). In order to select the appropriate resources, the students “have to evaluate the quality of the knowledge disseminated over the internet” (Engelbrecht et al., 2020, p. 826).

The situation is similar with the peer learning strategy. Online classes can help students find new learning partners but may also prevent them from meeting other students in traditional ways, such as meeting in the lecture hall. However, online meetings may have their drawbacks. Specific to mathematics, problems with notation arise, for example, when formulas or matrices cannot be communicated satisfactorily in digital chats (Boz & Adnan, 2017; Trenholm & Peschke, 2020).

### The COVID-19 pandemic and students’ learning

With the outbreak of the pandemic, many universities around the world changed to online teaching (Ní Fhloinn & Fitzmaurice, 2021a). In many places, the teachers created resources themselves rather than using existing resources (Hyland & O’Shea, 2021). With the increased use of asynchronous learning, students were expected to spend less time in live teaching and more time in self-directed activities (Alarfaj et al., 2021). It is therefore not surprising that the role that students' engagement played in the ability to achieve good learning outcomes increased when online teaching became prevalent (Büchele et al., 2021).

Accordingly, teachers considered it important to communicate frequently and clearly with students in online lessons in order to support students’ self-regulated learning (Ní Fhloinn & Fitzmaurice, 2021b). However, teachers' and students' assessments do not always coincide. For example, teachers seem to underestimate significantly the role that distractions at home play in a student’s ability to achieve success in an online learning environment (Radmer & Goodchild, 2021).

Students reported that communication with peers was very important (Kempen & Liebendörfer, 2021) but had become more difficult, and they reported increased isolation (Hyland & O’Shea, 2021; Radmer & Goodchild, 2021). Online collaboration was made possible through breakout rooms but was not satisfactory, in particular for pen-and-paper work (Lishchynska et al., 2021). Forums were rated even worse than work in breakout rooms. Students also collaborated privately by using WhatsApp, Discord, and other media. However, we do not know when and how such exchanges took place.

Surprisingly, one study revealed that many students prefer distance learning over in-class teaching (Hyland & O’Shea, 2021). But Reinhold et al. (2021) found that students with more promising affective and self-regulation behavior “reported a *higher* need for face-to-face social interaction at university—i.e., in-person collaboration with their fellow students and their lecturers” and “*less* preference for online learning formats after the pandemic” (p. 7, original emphases). These authors concluded that more promising students may have a greater need for direct communication. Alternatively, students with low learning success might benefit less from face-to-face formats because they are quickly overwhelmed and cannot engage in the discourse at all (Solomon, 2007). Thus, weaker students in particular may benefit from different materials that can be used as often as they like at their own pace (Kempen & Liebendörfer, 2021).

## The present study

### Background: online studies at a German university

Our study took place at a medium-sized German university where mathematics students and preservice secondary teachers attend the same online linear algebra course of lectures in the first semester. The lecture was initially offered online twice a week, but from the third week onwards, only once a week. It was not recorded. Students were asked to work through the course material themselves and to prepare questions for use in the lecture. The lecturer shared the lecture notes in advance and recommended additional resources (e.g., videos that she made or that were offered on the web). The lecture slides were made available to the students after the lecture.

To be admitted to the examination, students had to hand in solutions to weekly homework, earning at least 50% of the possible points. The tasks often required problem solving and proofs. Students were supposed to hand in their homework in pairs, and they were asked to find a partner on their own at the very beginning of the semester.

Students were offered a weekly full-class tutorial where solutions to the previous week’s homework were presented, and additional examples or remarks were discussed. They could work on additional tasks to prepare for the homework in tutor-led small-group tutorials with about 20 students each, in which individual questions could also be clarified. Neither tutorial was recorded. After the second lecture per week was eliminated, all live events were held at the beginning of the week between Monday and Wednesday morning. The homework had to be handed in on Thursdays at the beginning of the semester, then on Fridays from Week 5 onwards.

### Research question

Self-regulated learning is very important in mathematics studies. The literature indicates that the significance of self-regulation increases even more with online teaching as increased flexibility also means a greater need for structuring. It is an open question how students self-regulate their learning in fully online teaching during a pandemic. We thus sought to answer the exploratory, descriptive question: how do students regulate their learning and specifically their choice of resources and peer learning in university mathematics classes that are fully taught online as offered during the COVID-19 pandemic?

## Method

We present a case study with three cases. They are instrumental cases, according to Stake (1995). This means that the cases were not chosen because they were intrinsically interesting but to gain insights into general relationships. Case studies can illustrate how the abstract theories (in our case self-regulated learning) manifest themselves in specific situations. For example, they can illustrate what actions or resources may play a particular role in students’ learning during the pandemic. Although they cannot establish laws that govern student learning, case studies can show possible relationships and falsify assumptions (Flyvbjerg, 2006).

In terms of methods, there “are virtually no specific requirements guiding case research” (Meyer, 2001, p. 329). It is thus necessary to detail the methodological choices. Our research was guided by the framework of self-regulated learning and our research question about how students regulate their learning when learning online.

### Sample

All students in the linear algebra course at the chosen university were asked to participate in our study via the learning management system. Their anonymity was guaranteed, and they were free to leave the study at any time without experiencing disadvantages. The participants received an allowance of 10 euros for each report. At the beginning of the study, seven students participated. However, some students participated only sporadically or dropped out early. Therefore, we restricted our analysis to three students we call Colin, Bea, and Andy, who submitted at least 18 of the 28 reports; the other students had submitted one to 13 reports (less than 50%) and left the study before the semester was halfway over. Their cases will be summarized briefly in the results section.

Colin was a male mathematics major with average high school grades. He attended a special bridging course in school covering topics such as set theory and proving. Colin failed the examination but passed it one semester later. Bea was a female mathematics major who got her high school diploma with above-average grades more than 10 years before she started studying mathematics. She had brushed up on her mathematical knowledge with an online prestudy course. Bea had a part-time job during the semester. She qualified for the end-of-term examination and intended to switch to a computer science degree program. Andy was a male preservice higher secondary school teacher with average high school grades. Andy completed vocational training before going to university and missed the preparation course before the semester. He did not qualify for the end-of-semester examination but passed it one semester later.

### Design and procedure

Longitudinal research on students studying mathematics has so far shown that behavior and experience can change quickly (Liebendörfer, 2014). Therefore, we assessed students’ experiences several times during the semester to track such changes. The data had to be collected while students were away from university during the lockdown. For this purpose, we used audio diaries.

Audio diaries are suitable for capturing everyday life and also for collecting longitudinal data on personal and sensitive issues (Williamson et al., 2015). The data can get very close to the experienced situation, thus minimizing retrospective bias (Hislop et al., 2005; Williamson et al., 2015). Compared with interviews, “the diaries often offered a fuller picture of the day-to-day changes and fluctuations in experience” (Williamson et al., 2015, p. 30). By providing this fuller picture, they can depict contradictory experiences or behavior. Given the tension involved in the difficult secondary-tertiary transition and the possibility of dropout, we found audio diaries to be a suitable method for data collection. To direct the reports to relevant points, we used prompts that were similar to the questions used in a guided interview (Williamson et al., 2015).

Specifically, the students were asked to follow given guidelines to formulate an approximately 10-min self-report twice a week. The students could upload their reports directly to a university server or send them to the first author via WhatsApp. The first deadline was the Monday of Week 2 of the lectures.

The guide first asked for general learning behavior since the last report. Then the students were explicitly asked about the resources they had used. Next, they were asked to comment on times when they experienced success and perceived pressure. This was followed by communication with others. Finally, they were asked about mathematical content that they had understood particularly well or poorly. To motivate students to give more details, we asked them to rate individual items using a 6-point Likert scale and to explain their ratings. Our analysis focused on the explanations only. A slightly modified version of the guide was used from Day 11 onwards (see the Electronic Supplementary Material). The guide covered only students’ pressure but not their goals because prior research had shown that students only very superficially report learning goals and hardly ever report well-being goals, whereas they all report the goal of passing the examination when asked directly (as long as they continue to pursue their studies; Göller, 2020). We assumed that specific goals might be reconstructed from their answers to the questions about their general learning.

### Data analysis

The first part of our data analysis followed a qualitative content analysis (Kuckartz, 2019). In the first step, the data were coded along predefined categories corresponding to the questions in the guide, including pressure, general self-regulation, resources, and communication with peers. The coding was intended to ensure that statements about one topic that were part of an answer to another question were also taken into account. In many reports, each code was used only once (e.g., when students followed the guide perfectly). In the second step, we generated thematic summaries for each case and each category based on the coded text segments (Kuckartz, 2019, p. 194). The summaries for each person were organized into a thematic matrix with individual categories (rows) and points in time (columns).

This matrix allowed us both to identify relationships between the categories and to describe changes within categories in the third step. This step revealed different forms of regulation over time. Initially, for all students, their strategies, goals, and learning partners varied from report to report. Later, the variations decreased and were linked to certain events. The thematic summaries were therefore organized into phases. The first phase was considered completed as soon as they described a mode of working that was then followed consistently for at least three consecutive reports. Other phases were defined when there were significant changes in goals, strategies, or partners. To ensure consistent results, the interpretation was again compared with the transcripts, and the interpretation of the cases was discussed between the first two authors. Intermediate results were also presented to local communities of researchers for further discussion of our interpretations.

## Results

We present the three cases one after the other. The summaries are structured according to the phases we found. In each phase, we summarize the students’ general learning and their learning strategies and goals.

### Colin

#### Phase 1: Searching for a work mode

We organized Colin’s reports into three phases. In the first four reports (Phase 1), he expressed both high learning goals and performance goals and felt a great deal of pressure. He worked on his lecture notes, attended all the tutorials, and tried to complete all the tasks. Colin noted down questions whenever he had any and noted additional examples in his lecture notes in line with his high learning goals. By constantly changing his plans and starting over again trying to learn the material, Colin showed that his plans were not yet a good fit. We thus labeled this phase “searching for a work mode.”

Colin used traditional resources, in particular the lecture notes, to access the content. He used digital media (WhatsApp, Skype) to collaborate with peers, including his learning partner but also other students. Colin very early found that peer learning helped him monitor his understanding: “When I was asked things I then really noticed whether I had already understood or whether I was actually still having difficulties myself” (C1).

#### Phase 2: routinized work

Colin then started a 6-week-long phase of similar self-regulation we called “routinized work” (Phase 2). He attended all lectures and tutorials, and consistently reported the pattern of attempting to complete the tasks himself at the beginning of the week, then exchanging the solutions with his partner, finishing everything together on Wednesday at the latest, and writing it down. We concluded that Colin still had both high learning goals and high performance goals. He balanced these goals with well-being goals, for example, when he decided not to do any university work on a public holiday: “that's why the whole thing is somehow more relaxed this week […] Maybe I'll change my mind in this regard, if I'm too tempted then not to do much” (C7).

The structure provided by the lecture became apparent when the second weekly lecture was eliminated and the students were asked to watch videos that they could access from links embedded in the lecture notes instead: “You can now look at the videos in the script and so at any time. There is still a bit of structure missing, which I personally would have liked to have now” (C7).

Colin still mainly worked with the lecture notes. He exchanged information with his learning partner and sometimes a few other students via WhatsApp and especially Skype. It was surprising how Colin described a meeting in person that they once arranged:

Wednesday we met, this time in person. Yes, we discussed the tasks there. We were all not ready yet. So we all weren't done with the tasks yet, always just the one, two and part of the three, and did the rest together. Yes, that went pretty well, too. (C14)

In a 14-min report, Colin did not say a word about the difference between peer learning via online platforms or physically face to face. As we asked for students’ learning behavior and their experiences, we assumed that he would have reported details if this in-person meeting had made a big difference in his learning. We thus interpreted his report as an indication that collaboration via online platforms worked similarly well for him.

#### Phase 3: relaxation

We observed a significant change in Colin's self-directed learning when he was confident he could achieve the required score on the homework to be admitted to the examination: “I don't want to say we ran out of steam, but it's a bit quieter, if not too quiet” (C17). We called this third phase “relaxation.” Colin could achieve his first performance goal and immediately realized that because he was no longer feeling the pressure he used to feel, he did not do as much for his studies. Attending the live elements, Colin still had learning goals, but his self-study changed:

I still went to the lectures until the last day. […] The motivation has decreased a lot. With me, for example, it was now so that I have downloaded the things only on Monday at all. A bit, minimally I have looked at them only on Tuesday. On Wednesday, I briefly looked at it. […] And only on Thursday I calculated something concretely. We didn't discuss it until 6:00 p.m. in the evening, and it took us longer, until about midnight, until we were finished and handed it in. (C20)

Emphasizing his well-being, Colin reduced both his use of resources and peer learning. He planned to reengage more intensely soon by beginning to prepare for his examination.

The pressure is not so high right now [...] but yes, I have to start right now with the exam preparation. Then, I think, the pressure will be higher, [...]. Or, if I delay it too long, if that should happen, then in the area of very strong pressure. (C24)

The quote illustrates the tension between his performance goals and well-being goals. Although he knew he should startimmediately, he suspected that he might not be able to do much for a while yet.

#### Summary

Colin’s learning goals were closely aligned with his performance goals. He wanted to understand the content primarily if it was also important for passing the module. In addition, he actively managed his well-being goals. The time structure of the lecture helped him regulate his self-study. This aspect is evident in the relaxation phase, where he attended the live lecture but did not look at the self-study materials for a long time. For resources, he focused on the lecture notes and used digital media to interact with his peers. Digital peer learning worked equally well for him as did face to face learning.

### Bea

#### Phase 1: searching for a work mode

We organized Bea’s reports into five phases. In Phase 1, she found new partners and resources. As we did for Colin, we labeled this phase “searching for a work mode.” Similarly to Colin, Bea structured her learning along deadlines and dates because this helped her understand what had to be done by when. As she wanted to work on tasks early, she found it problematic to be assigned exercises that built on knowledge that was taught only after the assignment had been issued.

Initially, the pressure was low because Bea could easily meet the requirements. Like Colin, Bea specifically planned free time (e.g., a bicycle tour) to support her well-being.

In terms of resources, Bea also focused on the lecture notes. If she missed any basics, Bea looked them up in materials from the university’s bridging course. Bea soon used Discord to discuss assignments in a large group (at least 20 people). She could share her frustration and joy with the learning partner she found on Discord anytime if needed.

#### Phase 2: routinized work

In Phase 2, Bea had a consistent routine, using the same resources, mostly the lecture notes, and communicating with the same peers. As for Colin, we called this phase “routinized work.” Bea’s behavior indicated strong learning goals. She used the lecture notes as intended to prepare follow-up questions for use in the lecture and also attended the tutorials. She tried solving the exercises herself before she exchanged information with others, focusing on comprehension (learning goal) rather than on getting a solution (performance goal):

In the evening, it seems to be stabilizing that we do this every Tuesday. There was a meeting on Discord where we clarified questions about the homework. It also seems to be stabilizing in such a way that we no longer present any solutions at all but simply compare our approaches and clarify aspects where there are major difficulties in understanding. (B6)

Initially, Bea found the time structure to be reasonably good, but like Colin, she liked it less when the second lecture was eliminated. Soon after, Bea no longer found the time structure of the course to be helpful. They had the assignments for one week but received the final information needed to complete some tasks only in the lecture 2 days before the deadline. She found this to be too much time pressure. In addition, the structure of the week was not compatible with Bea's part-time job.

Unlike Colin, Bea chose resources other than the lecture notes when she wanted to understand a single concept (e.g., identity matrix) or she needed to solve a problem. She tended to use texts rather than videos as her resources. In any case, however, these resources had to help immediately, so she did not use Internet forums. Bea further preferred direct communication because she could then more easily clarify misunderstandings. Like Colin, Bea exchanged information with her learning partner but also with many people via Discord. It was ‘give and take’ for Bea, so she could build a stable learning group.

#### Phase 3: hospital

Phase 3 is specific to Bea. It occurred when Bea had to go to the hospital, so we called this phase “hospital.” Because everything was online, she could also study there. However, the WiFi was slow, so she initially missed the live events. Later, she was able to watch the lectures again. Her goals and collaboration with peers remained similar to the routinized work she had before going to the hospital and again after she was released.

#### Phase 4: shock and recovery

Phase 4 is also specific to Bea. She was falsely accused of plagiarism and then almost did not seem to care about anything except the accusation until it was withdrawn. Referring to her psychic state, we named this phase “shock and recovery.” Bea's learning partner had talked about their solution to a problem with another student using screen-sharing. This student had then submitted an identical solution, so that they all received no points for the paper because of suspected plagiarism. This overshadowed the entire week because her admission to the examination was in acute danger. Bea communicated a lot with the teaching team and with other students and hardly worked on the course material during this time. She did not join the meetings with peers on Discord except for personal meetings with her learning partner. This illustrates that Bea could only focus on her learning goals as long as the formal requirements for her performance goal of being admitted to the examination could be achieved. As the reproach was then dispelled, and Bea had also earned many points on the following homework, her admission to the examination was practically guaranteed.

#### Phase 5: relaxation

Phase 5 followed her recovery. Bea was sure to get admitted to the examination and now studied with less commitment, prioritizing her well-being. Similarly to Colin, we called this phase “relaxation.” Like him, Bea invested less time in her own preparation and revision of the materials but still attended the lectures and tutorials. She used the same resources as always, albeit somewhat less intensively. Her peer learning was limited to an exchange with her learning partner. She did not report meetings with other people on Discord during this phase.

#### Summary

Bea's case is similar in many ways to Colin's. She also pursued learning goals as long as the performance goals were achievable, but this pursuance dropped off significantly when the performance goals were achieved. Both also had weekly routines that aligned with the lecture time structure. For resources, lecture notes were also very important to her, but she supplemented them with other media at certain points. Online collaboration with peers worked very well. Bea benefitted specifically from online teaching, as she could continue studying at the hospital.

### Andy

#### Phase 1: Searching for a work mode

Andy's reports could be organized into five phases. In Phase 1, he changed partners and varied his resources as Colin and Bea did. We thus again labeled this phase “searching for a work mode.” Also similarily, Andy wanted to prepare and review all the lecture materials well and also work on the exercises well enough to get admitted to the examination. These desires indicated high learning goals and performance goals. Andy's first learning partner stopped studying mathematics in Week 2. Andy found a new partner who was already in a higher semester and was repeating the course. Andy could no longer complete the homework himself from Week 2 on, so he looked for further help on Discord. There, he mainly had solutions explained to him.

In Phase 1, Andy felt medium pressure from the homework. The given time structure was important to Andy. It helped him determine what he should have done or understood by when. Unlike Colin and Bea, however, Andy did not develop routines and often did not work, particularly when there was no deadline coming up. In Report 5, for example, Andy stated that he had done nothing for 2 days. Whereas Colin and Bea had their pauses planned deliberately, Andy seemed to prioritize well-being through in-the-moment regulation. This means that Andy's main issue was not that he felt too much pressure, but too little. He was afraid that he might not pass the module, used coping strategies and was dissatisfied with his lack of drive.

I looked at the homework sheet for tomorrow and understood relatively little or almost nothing, and I feel a bit of pressure because I need to do it soon and I don't understand it at all. On the other hand, if you're at home all the time, studying at your desk and not at university, it’s difficult to feel the pressure that motivates you to study a bit more. Because you're sitting in the same room where you sleep and watch Netflix and stuff. And I find it difficult to motivate myself to sit down and study for an hour and a half or two hours at a stretch, or to do something for university. (A3)

Andy used different resources than Colin and Bea did. In addition to links to videos that had been provided by the lecturer, Andy also used videos he had found on YouTube. Among them was a channel called “simpleclub” that presented information in a fun way but was often mathematically inaccurate and sometimes incorrect. He was occasionally annoyed that the lecture was not recorded because he could not always follow along and liked to watch things again.

Andy mainly communicated with his learning partner via WhatsApp and also exchanged information on Discord, mostly with other students who were also repeating the course. In the beginning, he also used an app that was popular among university students but was not specifically designed for mathematics. With this app, people could write anonymous posts that were displayed to other users within a radius of 10 km. This app was no longer mentioned from Phase 2 on. Andy preferred this app and Discord over the lecture’s online forum because the responses were faster. Compared to Colin and Bea, Andy used more different resources, but this seemed less satisfying and sustainable.

#### Phase 2: seriously trying

In Phase 2, Andy emphasized both his learning goal and performance goal of passing the course, but his activities showed little success and he started to compromise. Therefore, we called the phase "seriously trying."

I've been working through the lecture notes again for myself in the last two or three days, for one thing, and I've been making notes on it up to the rank of a matrix. […] In the tutorial, I didn't quite succeed, I didn't quite understand it, and I looked at it again after the tutorial, but I've postponed it for now, and I want to look at it again over the weekend. Furthermore, I did the homework with my learning partner. We both tried to solve the tasks, but we both had a hard time. (A6)

Andy prepared for and attended the lecture and later reviewed the lecture notes. He still watched videos but no longer reported using the simpleclub channel. Sometimes, Andy waited for the lecture and tutorials if something was unclear. He also attended the tutorials and reviewed the exercises after the small-group tutorial. On days without a fixed date, however, Andy often did not study: “[You should] definitely study a lot, preferably every day. Unfortunately, I usually lack the motivation to do that, but I definitely recommend it” (A12). Andy thus used coping strategies to support his well-being, to a much greater extent than Colin and Bea did.

In Report 10, Andy handed in his first incomplete sheet because he and his partner could not find anyone on Discord who could help. On the homework, he got less than 50% of the points this time. As his own performance fell short of the formal requirements, the pressure on Andy increased in Phase 2.

Despite his different self-regulation, like Colin and Bea, Andy had problems with the time structure when the second lecture on Thursday was cancelled. When he was required to structure longer periods of time himself, he often tried to cope by doing nothing.

#### Phase 3: surrendering

In Phase 3, Andy realized that he would not be admitted to the examination and gradually dropped the course. As he did not want to do this but did not see any reasonable alternatives, we called the phase "surrendering." Andy first prioritized performance goals over learning goals. He missed the full-class tutorial and focused on the homework instead, but he did not get enough points. He then increased coping by missing the small-group tutorial, stopping to work through the lecture notes completely, and no longer trying to understand everything. Finally, he also stopped attending the lecture.

Andy gradually stopped all his learning activities, but he did not make a new plan to follow. Only at the end of the surrendering phase did he plan to start learning again after the examination.

#### Phase 4: on hold

In Phase 4, Andy had no learning activities for linear algebra but planned to start again after the exam. Andy concentrated fully on the second module. We thus called this phase “on hold.”

#### Phase 5: restarting

We called Phase 5 “restarting.” Andy wanted to prepare for the upcoming semester, indicating learning goals and maybe some performance goals. He started reading the lecture notes, went through old notes, and solved the first problem on the first homework. Then he stopped again. In this phase, he had no contact with peers.

#### Summary

Andy initially had similarly strong learning goals and performance goals as did Colin and Bea. However, he failed to work consistently towards these goals. He had difficulty finding appropriate and sustainable strategies for himself. His various resources, his first and second learning partners, and his loose contacts on Discord did not help him study with routine and success.

### The four students who dropped from the study

We excluded from the analysis a student who submitted only one interview of four and a half minutes. Several parts were acoustically unintelligible and the student did not follow the guidelines for the interview. We now outline the cases of Dirk, Emil and Franz.

Franz was a male preservice higher secondary school teacher. We have 13 reports from him covering the first 7 weeks. In the first phase, he only worked together with his homework partner, with whom he felt no personal connection. Eventually, his partner stopped cooperating in the course. From then on, Franz no longer felt socially involved and finally dropped from the Linear Algebra course to be able to concentrate fully on his parallel course in mathematics. His case is similar to Andy's.

Emil was a mathematics major; from him we have 5 reports from the first 3 weeks. He mainly used lecture notes and Discord, where he was able to develop a regular work rhythm and felt socially involved. As far as he reported, his case is similar to Bea's.

Dirk was also a mathematics major. Reports from him are available only at the first and fifth time points. He used a variety of different resources, especially 'Google' for questions. He had a feeling and related concern that he could not make helpful contributions in his learning group. Similarly to Andy, Dirk did not report a working mode that eventually worked for him.

Franz, Emil and Dirk were as equally successful as the other three cases that we analyzed in this study. Dirk and Emil attained admission to the examination; Franz missed the necessary points on the homework.

## Summary and discussion

The aim of this study was to analyze how students learn when studying digitally during the COVID-19 pandemic. The research question was: “How do students regulate their learning and specifically their choice of resources and peer learning in university mathematics classes that are fully taught online as offered during the COVID-19 pandemic”?

Colin and Bea showed that the online mode could work well. As the COVID-19 pandemic required social distancing, this included working on homework together online. It was surprising to see that Colin did not report differences when studying with peers online or on a specific occasion studying in the same physical location. Bea specifically benefited from fully online teaching when she was in the hospital but also experienced specific disadvantages when a peer copied her solution after screen sharing. Andy’s case illustrates problems in building up routines, and in focusing on studying while being at home.

The regulation observed in three cases was similar in many respects to traditional mathematics studies in Germany. In particular, students’ goals were strongly oriented toward performance in homework (Göller, 2021). In line with findings from traditional studies, students felt strong pressure from the homework when learning online (Liebendörfer, 2018). This pressure is double-edged. It helped in choosing learning strategies for mastery of the content. As could be seen with Colin and Bea in their relaxation phase, without pressure both immediately decreased their engagement and prioritized well-being over learning. We thus assume that without the compulsory, graded homework, students would more strongly avoid learning difficult content, in favour of supporting their well-being (Boekaerts, 2011).

Compulsory homework appears to be primarily related to performance goals (Pekrun, 2006), at least when students only narrowly attain the required points. Then, students may start using coping strategies (Boekaerts, 2011; Göller & Rück, in print). When Bea perceived a threat to being allowed to take the final examination, the points got much more attention than the content. Andy prioritized performance goals over learning goals for a longer time, as it was more important for him to have a solution than to understand the content. This finding parallels findings from traditional teaching where working on the materials can turn into coping (e.g., students may copy their peers’ solutions; Göller, 2021; Gueudet & Pepin, 2018; Liebendörfer & Göller, 2016).

Looking at the specifics of online learning during the COVID-19 pandemic, Bea's visit in the hospital underlines the benefits of the high accessibility of online teaching. Our analysis revealed that the timing of the course's regular meetings had a double-edged effect. Andy needed regular meetings and deadlines in order to work regularly, whereas Bea was very organized and partly annoyed by the rigid requirements. Self-regulation has been shown to be more difficult in online learning environments (Kim et al., 2015; Trenholm & Peschke, 2020). As Andy’s case illustrates, these difficulties might arise more often because in the COVID-19 pandemic, students were studying at home and were surrounded by distractions from everyday life (e.g., platforms for streaming films; Boz & Adnan, 2017; Radmer & Goodchild, 2021). As well-being strategies are even more available in online digital learning environments, students need to develop new learning strategies to continue learning despite distractions.

All three students were disappointed after the second weekly lecture was eliminated. Changing the structure of the learning environment is challenging for students, as they must adjust their learning behavior by revising their prior plans. For Andy at least, live lectures and tutorials helped him structure his week better than the videos that replaced the second lecture. His case emphasizes that providing routine and structure has been an underestimated function of university teaching during the COVID-19 pandemic (Radmer & Goodchild, 2021). A weekly structure scaffolds self-regulation. However, this structure should be meaningful, avoiding such actions as handing out assignments that refer to knowledge from future lectures. Like Bea’s regular meetings on Discord, scheduled online study groups were found to provide not only opportunities to work with peers but also structure (Mac an Bhaird et al., 2021). This finding emphasizes the idea that a pandemic calls for professors to provide strong leadership in higher education. Marshall et al. (2020), for example, emphasized the need for clear directions and effective communication in the COVID-19 pandemic.

In terms of resources, timing turned out to be crucial again. We observed that when students seek help, an immediate response is very important. Therefore, forums—even when they are online—are of little help (see also Lishchynska et al., 2021). Andy’s choice of entertaining learning videos reveals that resource-related strategies may reflect students’ problems. Monitoring their learning, students might notice that they need other media to master the content. They regulate their choice of resources and use nontraditional media that might look more accessible. The intensive use of nontraditional media may thus indicate difficulties in learning the content (Kempen & Liebendörfer, 2021).

Contrary to the limitations reported in online collaborations in mathematics (Boz & Adnan, 2017; Trenholm & Peschke, 2020) but in accord with recent findings from the pandemic (Mac an Bhaird et al., 2021), the students in this study were able to exchange information well with peers. Maybe the technology was more advanced by that time or the COVID-19 pandemic (e.g., restrictions regarding social distancing) made the students more willing to engage with digital media, especially Discord. This tool offers forum structures and voice chats supporting both written and spoken language, which might both be important components of a fruitful collaboration. However, the exchange must be equal and thus sustainable in the long term. Digital dead ends (e.g., Andy’s app and help from people who probably could not handle higher content and also did not get anything in return) did not seem to work in the long run. The need to not only take information in fruitful collaboration could explain why the stronger students in particular want more face-to-face contact (Reinhold et al., 2021). In addition, trust must be achieved, as could be seen when Bea was suspected of plagiarism. Further, an immediate reaction to questions is guaranteed when there is face-to-face communication. Presumably, more social exchange in a physical place would have strengthened students’ commitment. It could also have helped to build routines and attract reliable exchange partners. Despite the problems that have been reported in online collaborations in mathematics (Lishchynska et al., 2021), the lecturer’s requirement that students work in pairs was very effective for the students in our study to form learning partnerships.

As an unexpected result, this in-depth longitudinal analysis shows that students' self-regulated learning goes through different phases. Some phases are more general (e.g., “searching for work modes”), and other phases are more individual (e.g., “hospital” in our study). A similar structure for self-regulation in university courses can also be assumed in traditional studies.

### Limitations

Due to its nature, our study can provide insights only into individual cases. A strong limitation is that presumably, only well-organized students volunteered for this type of study, and among them, we selected three students who had submitted almost all the requested reports. It would be important to know what self-regulation looks like for less-organized students or dropouts. In particular, students who dropped from the course in the first two or three weeks can hardly be covered with our approach. Because we paid our participants, we also might have affected not only their self-selection but also their learning. Our students were asked to reflect on their learning twice a week. Thus, further research should also try to analyze the less organized and less self-reflecting students as well as early dropouts who might suffer more from the need to more strongly self-regulate their studies during a pandemic. This might help in identifying more obstacles or helpful strategies for successful self-regulation. Whereas financial incentives are the most common way in qualitative research, researchers could also make clear the potential benefits of the research to others as an incentive (Robinson, 2014). In higher education, lecturers might require reflections as part of the homework or give bonus credits. The latter point might specifically motivate students who are at risk.

### Implications for research

Our results point to an under-researched field in online mathematics learning, which places higher demands on students’ self-regulation. As online pedagogy develops further (Engelbrecht & Harding, 2005), it needs to focus on structuring students’ learning processes. Our study revealed how seemingly small differences (e.g., replacing a live lecture with a set of videos) may substantially change students’ learning behavior and ability to self-regulate. This result complements existing research that found that teachers underestimate students’ needs for routine and structure when studying mathematics during a pandemic (Radmer & Goodchild, 2021). Andy’s case illustrated that students may be dissatisfied with their low motivation. Thus, the roles of meta-affect (DeBellis & Goldin, 2006) and meta-emotional knowledge (Corte et al., 2011) should be considered for support in self-regulation.

We further found that students might collaborate quite well even in the COVID-19 pandemic using online tools like Discord. As recent literature suggests problems with mathematical notation in digital chats (Boz & Adnan, 2017; Trenholm & Peschke, 2020), we should examine how new tools may support students’ oral and written communication. Given the specifics of our sample, we should investigate further if online collaboration during the COVID-19 pandemic worked for only some of the students.

Moreover, with the phases of self-regulation emerges a new finding that can presumably also be applied to classical mathematics studies. Future studies should investigate the stability of the phases and the question of whether the phases differ between successful and unsuccessful students (like Bea who quickly found a work mode and transitioned to routinized work, compared to Andy). This might help in identifying when exactly different self-regulatory demands arise.

### Practical implications

The apparent advantage of online learning of being available anytime and anywhere calls for more structure so that students can learn effectively. This includes a time structure that spreads learning activities out across the week. Lecturers should further help students choose their resources as the quality and content of media vary greatly. Lecturers could recommend resources or simply offer lecture captures. In addition, student collaboration should explicitly be encouraged, which has rarely been the case in traditional studies (Gueudet & Pepin, 2018). Learning partners, as used in this study, and scheduled online learning groups (Mac an Bhaird et al., 2021) may both be helpful to foster collaboration specifically during the COVID-19 pandemic with its restrictions on students’ collaboration.

Lecturers can further tailor their support to the different phases. For example, at the beginning of the studies, lecturers can help students find suitable learning partners and resources. Learning partnerships should involve ‘give and take’, in order to work in the long run. Similarly, students should be pointed to resources like YouTube channels that really support meaningful learning throughout the course. If students experience a relaxed phase, lecturers could motivate students to continue the work on the content before preparation for the examination begins.
